# Discrete-time survival analysis in the critically ill: a deep learning approach using heterogeneous data

**DOI:** 10.1038/s41746-022-00679-6

**Published:** 2022-09-14

**Authors:** Hans-Christian Thorsen-Meyer, Davide Placido, Benjamin Skov Kaas-Hansen, Anna P. Nielsen, Theis Lange, Annelaura B. Nielsen, Palle Toft, Jens Schierbeck, Thomas Strøm, Piotr J. Chmura, Marc Heimann, Kirstine Belling, Anders Perner, Søren Brunak

**Affiliations:** 1 Novo Nordisk Foundation Center for Protein Research, Faculty of Health and Medical Sciences, University of Copenhagen, DK-2200 Copenhagen, Denmark; 2 Department of Intensive Care, Rigshospitalet, Copenhagen University Hospital, DK-2100 Copenhagen, Denmark; 3 Clinical Pharmacology Unit, Zealand University Hospital, DK-4000 Roskilde, Denmark; 4 Department of Public Health, Section of Biostatistics, University of Copenhagen, DK-1014 Copenhagen, Denmark; 5 Department of Anaesthesiology and Intensive Care, Odense University Hospital, DK-5000 Odense, Denmark; 6 Department of Clinical Research, University of Southern Denmark, DK-5000 Odense, Denmark; 7 Department of Anaesthesia and Critical Care Medicine, Hospital Sønderjylland, University Hospital of Southern Denmark, Odense, Denmark; 8 Centre for IT, Medical Technology and Telephony Services, Capital Region of Denmark, DK-2100 Copenhagen, Denmark

**Keywords:** Outcomes research, Prognosis

## Abstract

Prediction of survival for patients in intensive care units (ICUs) has been subject to intense research. However, no models exist that embrace the multiverse of data in ICUs. It is an open question whether deep learning methods using automated data integration with minimal pre-processing of mixed data domains such as free text, medical history and high-frequency data can provide discrete-time survival estimates for individual ICU patients. We trained a deep learning model on data from patients admitted to ten ICUs in the Capital Region of Denmark and the Region of Southern Denmark between 2011 and 2018. Inspired by natural language processing we mapped the electronic patient record data to an embedded representation and fed the data to a recurrent neural network with a multi-label output layer representing the chance of survival at different follow-up times. We evaluated the performance using the time-dependent concordance index. In addition, we quantified and visualized the drivers of survival predictions using the SHAP methodology. We included 37,355 admissions of 29,417 patients in our study. Our deep learning models outperformed traditional Cox proportional-hazard models with concordance index in the ranges 0.72–0.73, 0.71–0.72, 0.71, and 0.69–0.70, for models applied at baseline 0, 24, 48, and 72 h, respectively. Deep learning models based on a combination of entity embeddings and survival modelling is a feasible approach to obtain individualized survival estimates in data-rich settings such as the ICU. The interpretable nature of the models enables us to understand the impact of the different data domains.

## Introduction

High quality tools for survival prediction would be valuable in the intensive care unit (ICU) where decisions must be taken swiftly based on massive amounts of information. Over the last 40 years, a lot of effort has gone into developing prognostic scores for the ICU setting to facilitate bedside assessment of disease severity and mortality risk^1–4^. However, most of the models applied in clinical settings are traditionally based on simple regression methods using a few routinely measured variables. Most models also suffer from poor calibration thus limiting the clinical use for patient-level predictions^1,5,6^. Simplistic in nature, many models only provide once-off static scores failing to leverage and integrate prior disease history and the many longitudinal data produced in the ICU. Although algorithmic bias is a genuine issue that must be handled^7^, better machine learning-based prognostication could aid clinicians and help overcome the potential bias in clinical judgment^8^.

Indeed, the ICU setting generates vast amounts of data in the form of high-frequency (e.g., ventilators, telemetric apparatus, infusion pumps) and low-frequency (e.g., manual observations, biochemical samples) data. Improving prognostic models requires the use of advanced methods that natively handle this multiverse of ICU data characterized by time series, static values, and free text from clinical notes. The data input is crucial to a model’s performance and usefulness, but so is the operationalisation of the outcome. Previously, most severity scores have used static outcomes such as the risk of in-ICU, 30-day, or 90-day mortality^9^. Such outcomes turn a continuous outcome into whether the patient is likely to pass away before or after an arbitrarily defined threshold, yielding neat but problematic binary classification problems^10^. Another limitation to this strategy is the changing nature of the cohort throughout the follow-up period. As time passes, patients pass away and the prediction problem at hand morphs in a way that escapes simplistic classification models. Modelling the survival profile directly, on the other hand, arguably resolves these shortcomings. The full survival profile is often of greater clinical interest than arbitrary thresholds. Further, the changing nature of the cohort is built into the discrete-time survival model, and by applying model explanation techniques we may quantify and visualize the drivers of the predictions at short-, mid- and long-term.

In this paper we present and validate a model that natively handles inputs from heterogeneous data sources, structured and unstructured alike, to produce longitudinal survival profiles, using a large cohort of Danish ICU patients. A key aim of this work was to render the model actionable, by displaying how different data domains affected survival at different phases of follow-up.

## Results

### Population characteristics

**Fig. 1:**
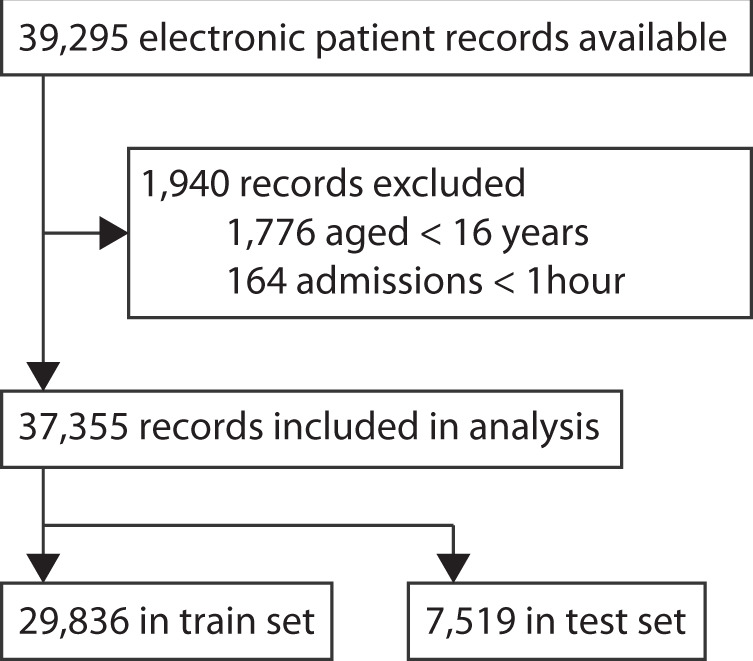
Illustration of the cohort selection procedure. The raw dataset contained electronic patient record data from 39,295 ICU admissions. 1940 records were excluded because the patients were too young (<16 years) or the admissions were too short (<1 h). The remaining 37,355 records were split into a training set (80%; *n* = 29,836 admissions) and a test set (20%; *n* = 7519 admissions).

30,763 patients had 39,295 ICU admissions in the Capital Region of Denmark and the Southern Region of Denmark between the 6th of September 2011 and the 19th of April 2018. Of these, 37,355 admissions (95.1%) of 29,417 patients (95.6%) were eligible for inclusion in the study (Fig. 1). We excluded 1940 admissions because the age of the patient was <16 years (1776 admissions, 4.5%) or the duration of the admission was <1 h (164 admissions, 0.4%). From this cohort, we allocated 20% (7519 admissions in 5900 patients) to the hold-out test set. The baseline characteristics of the patients are summarized in Table 1. For patients with more than one ICU admission, the data from the first admission was used in Table 1. Overall, the median age was 67 years and the ICU and 90-day mortality were 13.5% (3960/29,417) and 30.6% (9007/29,417), respectively.

**Table 1:** Baseline characteristics of ICU patients in the training and hold-out test sets.

	Training data set (*n* = 23,517)	Hold-out test set (*n* = 5900)
Patient demographics		
Age, years	67 [53–76]	66 [53–75]
Sex		
Female	10,047 (42.7%)	2541 (43.1%)
Male	13,470 (57.3%)	3359 (56.9%)
Co-morbidities		
Chronic heart failure	3768 (16.0%)	924 (15.7%)
Cirrhosis	894 (3.8%)	235 (4.0%)
AIDS	32 (0.1%)	8 (0.1%)
Metastatic cancer	1220 (5.2%)	279 (4.7%)
Haematological cancer	870 (3.7%)	263 (4.5%)
Cancer therapy	938 (4.0%)	236 (4.0%)
Admission category		
Medical	13,065 (55.6%)	3331 (56.5%)
Scheduled surgery	3134 (13.3%)	746 (12.6%)
Unscheduled surgery	7318 (31.1%)	1823 (30.9%)
Type of surgery		
Transplantation: Liver, kidney, pancreas	100 (0.4%)	39 (0.7%)
Transplantation: Kidney and pancreas, other	1 (0.0%)	0 (0.0%)
Cardiac surgery	324 (1.4%)	101 (1.7%)
Trauma	801 (3.4%)	205 (3.5%)
Neurosurgery	559 (2.4%)	136 (2.3%)
In-hospital location before ICU admission		
Emergency room	6882 (29.3%)	1640 (27.8%)
Other ICU	1953 (8.3%)	528 (8.9%)
Hospital ward, recovery unit, or operating room	14,682 (62.4%)	3732 (63.3%)
Length of hospital stay before ICU, days	1.0 [0.0–3.0]	1.0 [0.0–3.0]
Length of ICU stay, days	1.5 [0.8–4.1]	1.6 [0.8–4.0]
Number of admissions		
1 admission	19,272 (81.9%)	4830 (81.9%)
2 admissions	3081 (13.1%)	742 (12.6%)
3 admissions	755 (3.2%)	216 (3.7%)
≥ 4 admissions	409 (1.7%)	112 (1.9%)
Mortality		
ICU mortality	3154 (13.4%)	806 (13.7%)
In-hospital mortality	5715 (24.3%)	1458 (24.7%)
90-day mortality	7198 (30.6%)	1809 (30.7%)

Data are *n* (%) or median (inter-quartile range). For patients with multiple admissions the data provided below are from the first admission.

### Model performance

**Fig. 2:**
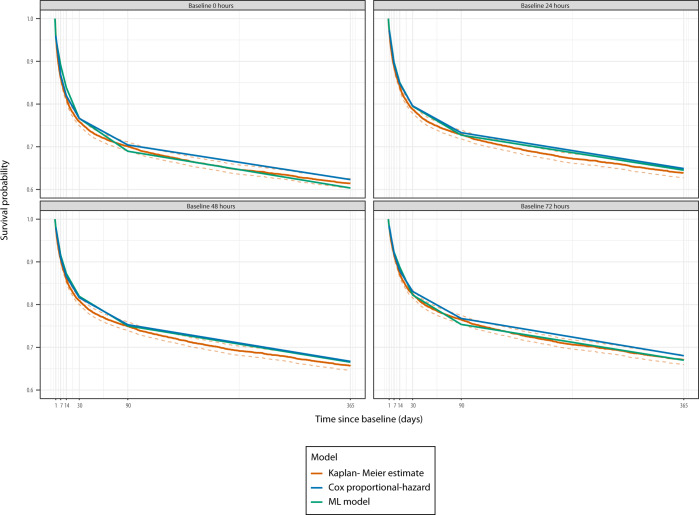
Comparison of survival predictions by our deep learning model and a Cox proportional-hazard model. The broken lines around the KM curves represents 95% confidence intervals. Each baseline (0, 24, 48, and 72 h) has its own curve, in a separate panel. The predictions for both models are close to the Kaplan-Meier estimate for the full hold-out test set.

**Fig. 3:**
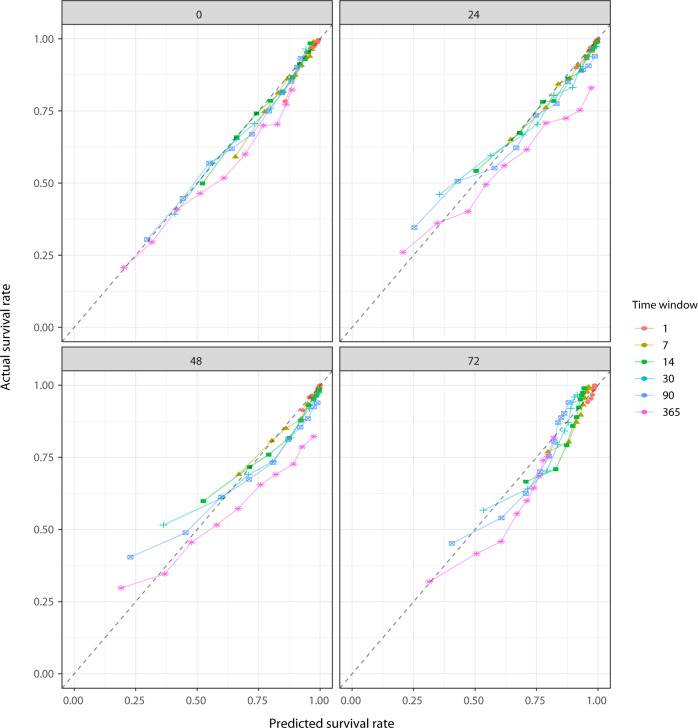
Calibration plots. Observed survival rates estimated by the Kaplan–Meier survival estimates method is plotted against predicted survival rates for baseline equal to 0 (i.e., “door-step”), 24, 48, and 72 h. Each prediction window (1, 7, 14, 30, 90, and 365 days have its own curve). The diagonal represents perfect calibration.

The model was based on discrete-time survival analysis, and in the following the performance of the model at each timepoint (1, 7, 14, 30, 90, and 365 days) is reported. Table 2 shows Harrell’s concordance index (C-index, a measure of discriminative ability) for the four ML models with different baselines, compared to conventional Cox models. For the Cox models only one C-index is shown per row because, due to proportional hazards, the C-indices are constant across prediction windows. For the models with baselines at 0, 24, 48, and 72 h after admission, the C-indices were between 0.69 and 0.73. For the Cox models, the C-indices were all 0.66. In Fig. 2, the Kaplan–Meier estimate for the internal validation (hold-out test set) cohort is presented with the predicted survival from our ML model and a Cox proportional-hazard model. Both models estimated the true survival very well at the cohort level. Overall, the models were reasonably calibrated (Fig. 3). The model with the 0-hour baseline (i.e., at ICU admission) exhibited the best calibration as its predictions lied close to the diagonal. For all of the four different baselines, predictions in the mid-range tended to be too pessimistic (predicted survival worse than observed).

**Table 2:** Comparison of the discriminative ability for machine learning (ML) based versus conventional Cox proportional-hazard models with baseline 0, 24, 48, and 72 h, respectively, after ICU admission.

		Prediction window (days)					
Method	Baseline	1	7	14	30	90	365
ML	0	0.72 (0.71–0.72)	0.73 (0.72–0.74)	0.73 (0.72–0.73)	0.73 (0.72–0.73)	0.73 (0.72–0.73)	0.73 (0.72–0.73)
ML	24	0.71 (0.71–0.72)	0.71 (0.71–0.72)	0.71 (0.70–0.72)	0.71 (0.71–0.72)	0.72 (0.71–0.72)	0.72 (0.71–0.73)
ML	48	0.71 (0.70–0.72)	0.71 (0.70–0.72)	0.71 (0.70–0.72)	0.71 (0.70–0.72)	0.71 (0.70–0.72)	0.71 (0.70–0.72)
ML	72	0.70 (0.69–0.71)	0.69 (0.68–0.70)	0.69 (0.68–0.70)	0.69 (0.68–0.70)	0.69 (0.68–0.70)	0.70 (0.69–0.70)
Cox	0	0.66 (0.66–0.66)					
Cox	24	0.66 (0.66–0.66)					
Cox	48	0.66 (0.66–0.66)					
Cox	72	0.66 (0.66–0.66)					

The concordance index is shown for each prediction window with 95% CIs in parentheses.

### Explainable prediction model

**Fig. 4:**
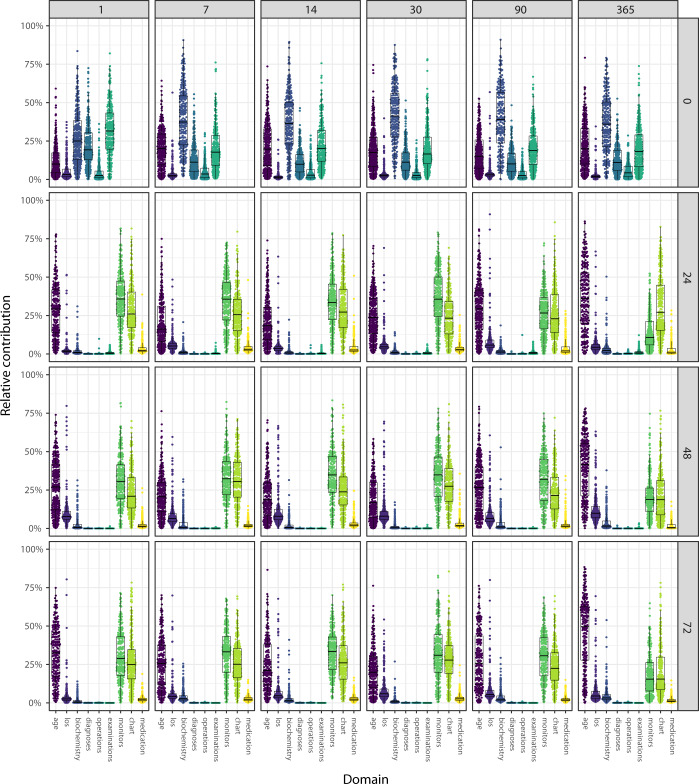
The impact of input domains on survival predictions. Each dot represents the relative contribution from a data domain to a given prediction for one patient. LOS = Hospital length of stay before ICU admission. Each column and row correspond to a specific prediction window (1, 7, 14, 30, 90, and 365 days) and baseline (0, 24, 48, and 72 h), respectively. In the figure the centre lines denote medians, bounds of boxes represent 25th (Q1) and 75th percentiles (Q3). Whiskers denote nonoutlier data range defined by Q1 − 1.5 interquartile and Q3 + 1.5 interquartile range.

Figure 4 illustrates the relative impact of the different input domains across the time windows and baselines on the predictions. For the “door-step” model (baseline = 0 h), which was only trained using information available before the ICU admission, the biochemistry domain had the largest impact; the medication also appeared informative. When the data captured in the ICU was subsequently introduced for models with a baseline > 0 h, this new information seemed to dilute the “signal” from the patient’s medical history. For all models and all prediction windows, the age at admission was one of the most important features.

## Discussion

In this study, we have presented an explainable deep learning model enabling integration of heterogeneous data sources such as medical history, free-text nursing documentation, and vital signs without the need for extensive pre-processing and feature engineering. The model provides individualized, discrete-time survival estimates for ICU patients. The survival model has time-varying hazards, and therefore overcomes the proportional-hazards assumption which, in combination with its interpretable nature, provides the opportunity to investigate how the impact of features affects early versus longer term mortality.

Using ML for prognostication in ICU patients is an active area of research^11^. To our knowledge, our models are the first to leverage the combined power of entity embeddings (allowing integration of heterogeneous data), a recurrent neural network architecture (appropriate for dynamic predictions with time-series data) and time-to-event outcome operationalisation (a prudent approach preferred over more simplistic binary classification models). Entity encoding is uncommon in healthcare-related studies although applications start to emerge, in general patient^12^ and ICU^13,14^ populations.

A study from 2019 by Nielsen et al. found that ML models based solely on previous disease history performed as well as mortality scores in clinical use. This model used medical history, but in a less principled way than ours, and used a simple multilayer perceptron as the prediction engine^15^. Second, the study by Thorsen-Meyer et al. from 2020 (serving somewhat as a precursor for this study) used an LSTM to predict the 90-day mortality in ICU patients and found very satisfactory performance in a relevant external validation set^16^. Third, a recent study by Pattalung et al. from 2021 used American data (MIMIC III, MIMIC IV, eICU) to develop a prediction model methodologically and scope-wise quite similar to that of Thorsen-Meyer et al.^17^.

The common denominator of these, and to the best of our knowledge all other published studies in the realms of ML-based prognostication in ICU patients, is their reliance on structured input data and binary classification-styled outcome. In prediction modelling perhaps the most prominent benefit of structured input data is the ability to take a model trained in one dataset and apply it in another, for validation or as part of clinical decision support. They do suffer from at least three potential problems: creating and, not least, maintaining structured data are complex and costly; their quality might decline due to data drift and changing coding practices (but using the same coding scheme); and the structured data in target contexts (in which the model would be deployed) may use different vocabularies, requiring some way to bridge these vocabularies and somewhat undermining the benefits of structured data in the first place. Entity embeddings can help overcome these challenges although they come with their own, as discussed below.

We used a novel technique for using “all kinds of data” as inputs, by first turning structured and unstructured data into tokens, which are then embedded into a dense representation and used as input in the survival model. This technique has several advantages. First, the need for feature engineering and data pre-processing is substantially reduced. Second, the model learns important trends by itself and very different data types (such as free text, static structured data, and high-frequency, waveform-like data from e.g., monitors) can natively be used as inputs together. Third, we use a loss function based on a log-likelihood function built to handle right-censoring. This allows for follow-up times substantially longer than the norm in studies of survival in ICU patients and compensates somewhat for the inability to extrapolate. Fourth, due to the representation of the input data, the model can embed input data from diverse EHR systems, regardless of the underlying data model. In this way, the model can learn, for example, that 100 millimetres of mercury is the equivalent to 13.3 kilopascals.

The study also has limitations. First, splitting follow-up time is arguably less optimal than a parametric survival model (e.g., a Weibull regression^18^) and precludes predicting beyond the follow-up time (365 days in this study). However, its non-parametric nature avoids distributional assumptions made by other methods, including those extending Cox regression^19^. Second, the log-likelihood assumes non-informative censoring (i.e., independent of the risk of the outcome^20^), which we find realistic given the population-wide coverage of the data and essentially no dropout except at the end of follow-up and due to migration. Were this assumption invalid, an informative censoring process could be built into the model, e.g., with a competing risks approach^21^. Finally, we did not validate the model in an external data set, nor did we assess the perspectives of clinicians, relatives, or the patients themselves. We forewent external validation for two principal reasons. First, the genuine clinical utility of any prediction model hinges entirely on its performance in the target population and, in our case, appropriate external validation data were unavailable as they would need to be in Danish (due to the model’s using entity embeddings) and collected prospectively, preferably in the context of a clinical trial. One could (re)train the models in e.g., MIMIC or eICU data to gauge if the method (not the models per se) transports to other data sources, but that was beyond the scope of this study. Second, such a prospective clinical trial should preferably only be initiated after the model has been scrutinized by peers to ascertain it be pertinent and reasonable.

In conclusion, we present an explainable deep learning model which can provide personalized survival time analysis for ICU patients. The model incorporates all available data with a modest need for pre-processing and without any requirements for cleaning.

## Methods

### Data and variables

In this study, we retrospectively collected electronic patient record (EPR) data from patients admitted to one of 10 mixed medical and surgical ICUs in the Capital Region of Denmark and the Region of Southern Denmark covering the period 6th of September 2011 through 19th of April 2018. Data were excluded for patients younger than 16 years of age and patients without available outcome data due to missing national identification number (NIN; all Danish citizens receive a NIN at birth or immigration). The data set was augmented by information from the Danish National Patient Registry (DNPR) and the Danish Civil Registration Registry (Danish: Det Centrale Personregister, CPR). The clinical information from the EPRs comprised different modalities. Biochemical data were extracted from two clinical laboratory information systems: BCC (Region of Southern Denmark) and Labka II (Capital Region of Denmark). The DNPR is a nation-wide registry containing information about encounters with the Danish hospital service. It includes information about admissions, examinations, diagnoses and treatments, all encoded in the Health Care Classification System (Danish: Sundhedsvæsenets Klassifikations System, SKS)^22^. In the DNPR disease and surgery codes observe the Danish adaption of the 10th revision of the International Classification of Diseases (ICD-10) and of the Nordic Medico-Statistical Committee Classification of Surgical Procedures (NOMESCO) used in Denmark since 1994 and 1996, respectively^23^. CPR contains information on all individuals who have been living in Denmark and registered in a Danish municipality after the 2nd of April 1968^24^. ICU clinical data were extracted from Critical Information System® (CIS, developed by Daintel, Copenhagen, Denmark, now acquired by Cambio Healthcare Systems). CIS is an EPR system customized for ICUs to store demographic and high-frequency data collected from equipment such as monitors, ventilators, and infusion pumps.

### Data domains and pre-processing

**Fig. 5:**
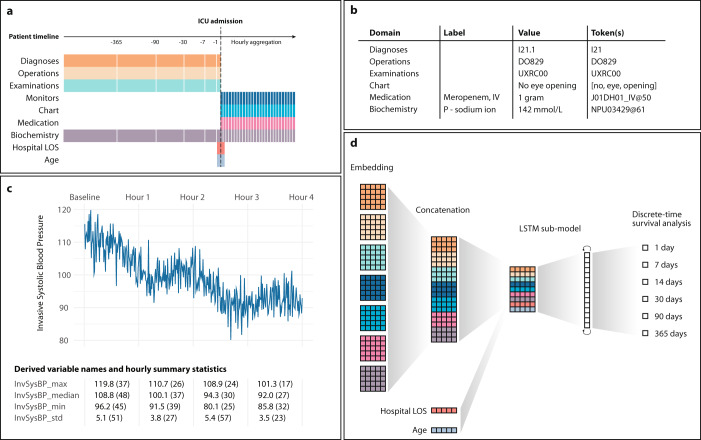
A schematic illustration of data processing and model architecture, inspired by natural language processing with input data cast into tokens. **a** The nine input data domains and the discretisation of time into windows (represented by rectangles) with greater granularity after ICU admission than before. **b** Tokenisation of six domains; for example, a three-word note in the chart yields three distinct tokens and 1 gram Meropenem (with ATC code J01DH01) administered intravenously is the 50-percentile in our data and so yields the J01DH01_IV@50 token. **c** This panel uses fictive recordings to exemplify the aggregation of high-sample-rate and high-frequency data to hourly summary statistics (maxima, minima, medians, and standard deviations) with their corresponding percentiles in parentheses; as such, one resultant token would be InvSysBP_max@37. **d** To make the model handle this data representation, the first layer consists of a separate embedding space for each input domain, which is updated during training of the model. *age* and *length of hospital stays before ICU admission* (Hospital LOS) are added deeper in the model as numerical values. The output nodes constitute a dense (linear) layer, in which each node yields the predicted probability of surviving the corresponding window (e.g., output node 1 ~ probability of surviving the first day and output node 2 ~ probability of surviving from days 2 through 6).

Data were divided into seven data domains based on their origin: medical history, surgical history, examination history (e.g., X-ray examinations), high-frequency data from ICU equipment, nursing chart (information entered manually in the EPR system, including free text), medications, and lab values. In addition, we added as features age and length of hospital stay (LOS) before ICU admission yielding a total of nine input domains (Fig. 5a). Medical history was represented as ICD-10 codes at block level, while surgical history and examinations were fed to the model as the full NOMESCO and SKS codes, respectively (Fig. 5b). Regarding the data from ICU equipment, chart information, medication, and lab values, we included all variables that appeared at least once in all departments, resulting in 64, 206, 1448, and 738 variables, respectively.

The high-frequency and other numerical data potentially collected more than once per hour data were aggregated to hourly summary statistics (minima, maxima, medians, and standard deviations) to make them compatible with data from the other domains, and to make the data fit in memory during calculations. In this way, each high-frequency data input gave rise to four features, for example InvSysBP_max, InvSysBP_median, InvSysBP_min and InvSysBP_std for the input variable *invasive systolic blood pressure* (Fig. 5c). Product names and names of biochemical analyses were replaced with ATC and NPU codes, respectively (Fig. 5b)^25,26^.

### Model components

The model had three key components, each of which we detail in the following: entity embedding of input features, long short-term memory (LSTM) sub-model, and the survival analysis output (Fig. 5d).

### Entity embedding and concatenation

To feed heterogeneous data into the model, we used entity embeddings^27^ (inspired by word embeddings in natural language processing^12,13,28^) based on so-called tokens, each representing data from an event in a patient’s timeline. These embeddings have been introduced into the machine learning field because they generally perform better than one-hot data encodings. They represent categorical variables in a compact and continuous way that can add informative relations between feature values. Specifically, the entity embedding serves as a way to map categorical data (the tokens) to a continuous form, in this case a d-dimensional embedding vector (i.e., a coordinate in a d-dimensional Euclidian space). The tokens came about in three different ways, depending on their origin (Fig. 5b, c). First, for numerical data, the tokens were created by combining the variable name with the percentile of the numerical value. Second, textual data were split into words each of which became one token. Third, for codified inputs (e.g., medical history) the codes became the tokens.

Within each domain, the collection of unique tokens constituted that domain’s so-called vocabulary whose size is the number of unique tokens therein. Generally, embeddings serve to represent the original data in fewer dimensions to elicit latent information and relationships not necessarily discernible in the original data. Thus, finding the right embedding size is a trade-off: we seek an embedding that captures all the information we need in as few dimensions as possible. If the embedding becomes too small, however, the data are squeezed too much so pertinent, latent information is lost, hurting the overall model performance.

Because there is no obvious way to specify the best embedding size a priori, we estimated this with a hyperparameter α_d_ (the embedding coefficient) determining heuristically, together with the vocabulary size V, the embedding size as *D* = 6α_d_*V*^1/4^^12^. So, for example with an embedding coefficient of 1, the embedding size would be 6 × 1 × 10,000^1/4^ = 60 for a vocabulary of size 10,000 (166-fold reduction is dimensionality) and 6 × 1 × 1000^1/4^ ≈ 34 for one of size 1000 (30-fold reduction).

Event temporality was operationalised as discretized timestamps: prior events were labelled as occurring <1, 2–7, 8–30, 31–90, 91–365, or >365 days before baseline whereas events after baseline were timestamped on an hourly basis (Fig. 5a). In this way, for example, within each domain embedding vectors of tokens observed <1 day before baseline were concatenated, so were the embedding vectors of tokens observed in the first hour of ICU admission, and so forth. This data representation condensed the parameter space to optimize model robustness and allow the model to learn abstract, high-level relationships between data points with no manual feature engineering. The entity embedding weights were trained jointly with the rest of the model.

### LSTM sub-model

To allow complex interactions and temporal patterns to be considered, we used an LSTM network as a sub-model to connect the embedded input features to the outcome survival predictions. LSTM networks are a specialized type of recurrent neural networks that continuously update their parameters as data accrue and extract temporal patterns from the input data^29^. This defining quality makes them suitable for time-series prediction tasks. The power of LSTM networks comes at a cost as they have many parameters. Training LSTM models requires large data sets and a somewhat specialized computing setup to obtain reasonable run-time.

### Survival analysis

Many approaches to survival analysis—both classic and deep learning-based—assume proportional hazards, an assumption seldom evaluated and often violated. To overcome this assumption, we chose a non-parametric discrete-time model in which follow-up time is divided into time windows, each with its own hazard, where the model learns survival in all windows jointly. This multilabel approach allowed the model to share information between the output nodes to fully exploit the data^30^. We applied a tailored loss function to handle right-censored patients, i.e., patients who do not experience the event in the observation period, either because they leave the cohort (due to e.g., emigration) or the follow-up period ends (after 365 days in this study); because death is inevitable, the observation period is shorter than the risk period for survivors, and so they are right-censored^20,31^. Indeed, time-to-event analyses are appropriate exactly when the risk period extends beyond the observation period.

The loss function was based on the log-likelihood^32^: the probability of dying in a given time window was the probability of surviving up to and actually dying in that time window. Censored patients contributed with the probability of surviving only as far as they were retained. We used the operationalisation of Gensheimer et al.^33^ and here build some intuition around the log-likelihood for a given patient, eq. (13) in Gensheimer et al.,

1 $$\ell = \mathop {\sum}\limits_{i = 1}^n {\left[ {\log \left( {1 + {\rm{surv}}_s^{\left( i \right)} \cdot \left( {{\rm{surv}}_{\rm{pred}}^{\left( i \right)} - 1} \right)} \right) + \log \left( {1 - {\rm{surv}}_f^{\left( i \right)} \cdot {\rm{surv}}_{\rm{pred}}^{\left( i \right)}} \right)} \right]}$$

where *n* is the number of windows, and surv_*s*_ and surv_*f*_ are indicator functions designating whether the patient survived or died during the *i*’th window (See Supplementary Fig. 1). Patients who were right-censored in the second half of a time window were considered to survive in that time window; otherwise, they were considered to have survived the preceding time window. Because ${\rm{surv}}_{\rm{pred}}^{(i)} = 1 - {\rm{hazard}}_{\rm{pred}}^{(i)}$ is the prediction of the *i*’th output node, the two terms inside the sum above can each take one of two values for a given output node,

2 $$\log \left( {1 + {\rm{surv}}_s^{\left( i \right)} \cdot \left( {{\rm{surv}}_{\rm{pred}}^{\left( i \right)} - 1} \right)} \right) = \left\{ {\begin{array}{*{20}{c}} {\log \left( {{\rm{surv}}_{\rm{pred}}^{\left( i \right)}} \right)} \\ 0 \end{array}{{{\mathrm{when}}}}\;{{{\mathrm{patient}}}}\;{{{\mathrm{survived}}}}\;{{{i}}}^\prime {{{\mathrm{th}}}}\;{{{\mathrm{window}}}}\;{{{\mathrm{otherwise}}}}} \right.$$

3 $$\log \left( {1 - {\rm{surv}}_f^{\left( i \right)} \cdot {\rm{surv}}_{pred}^{\left( i \right)}} \right) = \left\{ {\begin{array}{*{20}{c}} {\log \left( {{\rm{hazard}}_{\rm{pred}}^{\left( i \right)}} \right)} \\ 0 \end{array}{{{\mathrm{when}}}}\;{{{\mathrm{event}}}}\;{{{\mathrm{in}}}}\;{{{i}}}^\prime {{{\mathrm{th}}}}\;{{{\mathrm{window}}}}\;{{{\mathrm{otherwise}}}}} \right.$$

That is, if a patient died in a time window, the log-likelihood was the logarithm of the predicted hazard (risk of dying) of that window, and if not, the logarithm of the predicted chance of surviving that window.

The loss function for a given window was the negative of the sum of all patients’ log-likelihoods for that window. The seemingly strange behaviour of zero loss in windows after the patient died or was censored, regardless of the predicted survival probability, ensures that losses were only back-propagated for patients who actually contributed to the window in question.

### Model development

ICU patients were split into a training set and a hold-out test set. Patients born in the first 6 days of a month were assigned to the test set, yielding about 80% for training and 20% for testing. We used all admissions of the included patients. Using data from two distinct geographical regions—each with their own hospitals and patient populations—it may seem more prudent to split by region instead of date of birth. This would, however, cause information to leak between the sets because some patients were transferred between regions, rendering disentanglement impossible and thereby compromising the independence of the test set.

We used a five-fold cross validation scheme during training to select hyperparameter values and model architecture^34^. Cross-validation folds must be independent, so that e.g., a patient’s medical history is not used for both training and validation, which would undermine the independence of the validation set. Thus, the cross-validation folds were created at the patient level, allocating all admissions pertaining to a given patient to the same fold^30^.

We used *hyperopt*^35^ to optimize the hyperparameter settings, using a random search of the hyperparameter space constituting both predefined and variable hyperparameter values. We sought to optimize on these hyperparameters (discrete values in round brackets and uniform distribution boundaries in squared): recurrent layer design (LSTM, GRU), number of hidden units in recurrent sub-model [128, 384], recurrent sub-model dropout rate [0.1, 0.8], dropout rate [0.1, 0.8], L2 regularization parameter [0.0001, 0.1], embedding coefficient [0.2, 2], padding percentile [90%, 98%]. Increasing number of units in the LSTM sub-model made it more flexible but also more prone to overfitting which, in turn, was countered by dropout and regularization. For each epoch, dropout randomly removed a proportion of units from the network, and a larger regularization parameter put greater penalty on more complex models (i.e., favours simpler ones). The padding percentile was related to how many notes were used, since including more notes required more padding, see Supplementary Fig. 2.

Models were trained with the following different baselines; 0, 24, 48, and 72 h after ICU admission, respectively. Patients were only included in the corresponding training or test data set if they were alive at the baseline.

### Explainable prediction model

To get a better understanding of the model’s predictions, and to mitigate the issue of “black-box” predictions, we applied the Shapley Additive exPlanations (SHAP) algorithm to obtain local, post hoc explanations^36,37^. The SHAP methodology is a model-agnostic approach, that allowed us to elicit the contribution of a single feature contributes to the model’s overall predictions. The method is based on Shapley values from cooperative game theory^38^, expressing how much each feature, on its own and in concert with all other features, contributes to the difference between the actual prediction and the cohort-level mean prediction.

### Discrimination and calibration

The performance metrics were all computed in the hold-out test set. To gauge the discriminatory performance, we used the time-dependent concordance index (*C*^*td*^)^39^, a modified version of the conventional C-index, which in turn is a natural extension of the AUROC to survival analysis that takes censoring into account^40^. The C-index of a model is the fraction of all pairwise survival time predictions that are concordant. Two predictions are concordant if the patient with the highest predicted risk score has the shortest time-to-event. We used the original *C*^*td*^ foregoing a proposed modification to handle tied survival predictions^41^ because we assumed the risk of ties to be too low to warrant the added computational costs. To construct 95% confidence intervals (CI) around the performance estimates, we applied bootstrapping using 1000 samples with replacement of mortality prediction probabilities.

We assessed calibration using visual and numerical evaluation of the calibration plots for each window^42^ and based calibration plots on predicted survival and the Kaplan–Meier (KM) estimates. For each follow-up time and each baseline, we grouped patients by deciles of predicted survival probability and computed the mean KM estimate within each group. The former was plotted on the *x*-axis and the latter on the *y*-axis to construct the calibration plot.

A traditional Cox regression model was fitted on the training dataset with age, sex, and hospital LOS before ICU admission as predictor variables. The performance of this Cox model on the hold-out test set was reported for comparison.

### Ethics

This study was approved by the Danish Patient Safety Authority (3–3013–1723 and 3–3013–1731), the Danish Data Protection Agency (DT SUND 2016–48, 2016–50, and 2017–57) and the Danish Health Data Authority (FSEID 00003724 and FSEID 00004758). This paper adheres to relevant items in the Transparent Reporting of a multivariable prediction model for Individual Prognosis or Diagnosis (TRIPOD) statement^42^.

### Reporting summary

Further information on research design is available in the Nature Research Reporting Summary linked to this article.

## Supplementary information

Supplemental Material

Reporting Summary

## Data Availability

Due to national and EU regulations the data accessed in this paper cannot readily be shared to the wider research community. However, data can be made available for use in secure, dedicated environments via application to the Danish Patient Safety Authority (https://stps.dk/da/ansvar-og-retningslinjer/patientjournaloplysninger-til-forskning) and the Danish Health Data Authority (https://sundhedsdatastyrelsen.dk/da/english/health_data_and_registers/research_services).

## Code availability

Python code used to train the models in this study can be accessed at GitHub (https://github.com/daplaci/ICUsurvive).
