# Supplementary material for: Discrete-time survival analysis in the critically ill: a deep learning approach using heterogeneous data
Source: NPJ Digit Med. 2022 Sep 14;5:142. doi: 10.1038/s41746-022-00679-6 (PMC9474816; doi:10.1038/s41746-022-00679-6)
Supplement: Supplementary file 1 — Supplemental Material [file 41746_2022_679_MOESM1_ESM.pdf]

Discrete-time survival analysis in the critically ill: a deep learning approach using heterogeneous data

Hans-Christian Thorsen-Meyer[1,2], Davide Placido[1], Benjamin Skov Kaas-Hansen[1,3,4], Anna P Nielsen[1], Theis Lange[4], Annelaura B Nielsen[1], Palle Toft[5,6], Jens Schierbeck[5,6], Thomas Strøm[5,6,7], Piotr J Chmura[1], Marc Heimann[8], Kirstine Belling[1], Anders Perner[2], Søren Brunak[1]

[1]Novo Nordisk Foundation Center for Protein Research, Faculty of Health and Medical Sciences, University of Copenhagen, DK-2200 Copenhagen, Denmark

[2]Department of Intensive Care, Rigshospitalet, Copenhagen University Hospital, DK-2100 Copenhagen, Denmark

[3]Clinical Pharmacology Unit, Zealand University Hospital, DK-4000 Roskilde, Denmark

[4]Department of Public Health, Section of Biostatistics, University of Copenhagen, DK-1014 Copenhagen, Denmark

[5]Department of Anaesthesiology and Intensive Care, Odense University Hospital, DK-5000 Odense, Denmark

[6]Department of Clinical Research, University of Southern Denmark, DK-5000 Odense, Denmark

[7]Department of Anaesthesia and Critical Care Medicine, Hospital Sønderjylland, University Hospital of Southern Denmark, Denmark

[8]Centre for IT, Medical Technology and Telephony Services, Capital Region of Denmark,

DK-2100 Copenhagen, Denmark

Corresponding author:

Søren Brunak, e-mail: soren.brunak@cpr.ku.dk, phone: +45 20 67 24 77.

Contents

- Supplementary Figure 1: Illustration of how label data are derived.
- Supplementary Figure 2: Padding of token sequences.

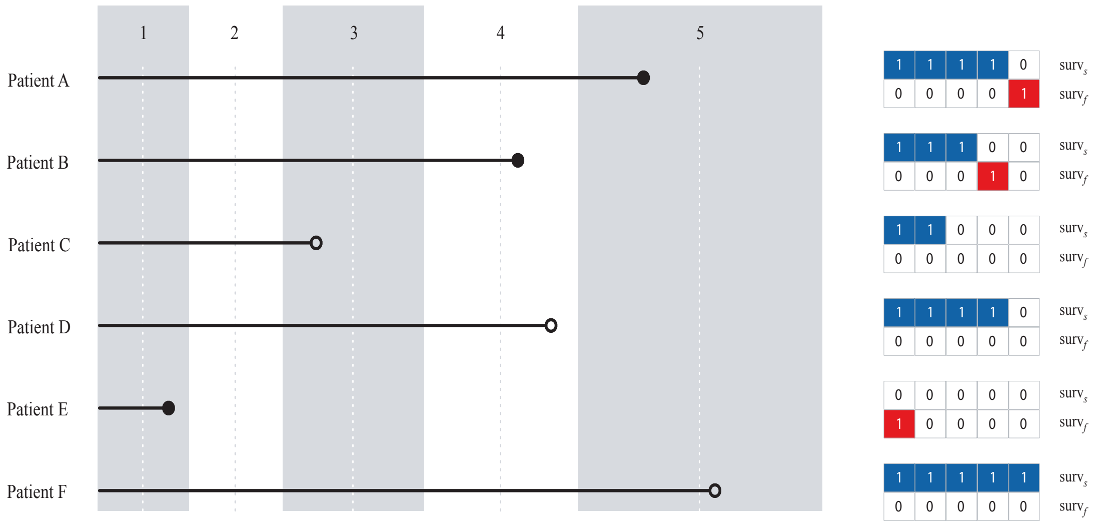

Supplementary Figure 1: Illustration of how label data are derived. The figure shows six fictive patients who are followed for varying numbers of windows. Windows grow increasingly wider. Filled circles represent events (death), hollow circles right-censoring; dashed vertical are mid-window marks. When patients are censored in the first half of a window, they are considered to have survived up to and including the preceding window; otherwise they are considered to also survive the window during which they are censored. *s*: survival throughout the window, *f*: failure (= event, death) during the window.

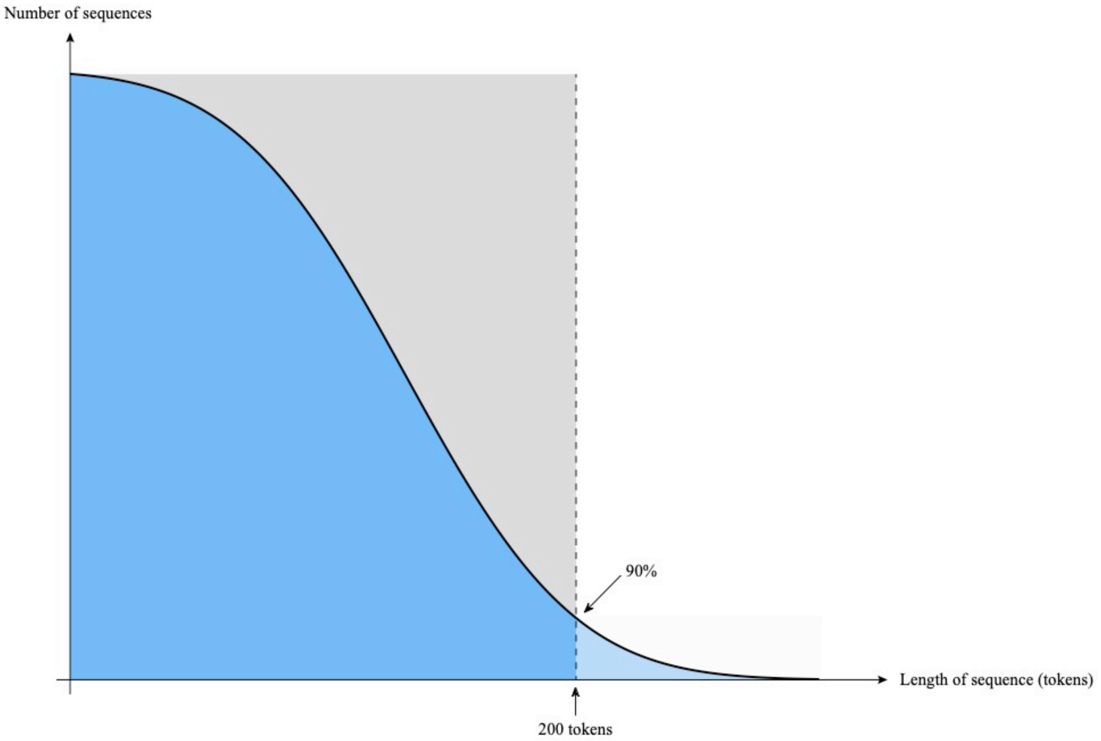

Supplementary Figure 2: Padding of token sequences. This fictive example uses a 90% padding percentile. In this example, this corresponds to a length of 200 tokens because 90% of the sequences have 200 tokens or less. Shorter sequences are padded (grey area), longer ones are cropped at 200 tokens (light blue area). Hence, a larger padding percentile will cause more padding but less cropping.
