# Supplementary material for: Discrete-time survival analysis in the critically ill: a deep learning approach using heterogeneous data
Source: NPJ Digit Med. 2022 Sep 14;5:142. doi: 10.1038/s41746-022-00679-6 (PMC9474816; doi:10.1038/s41746-022-00679-6)
Supplement: Supplementary file 2 — Reporting Summary [file 41746_2022_679_MOESM2_ESM.pdf]

nature portfolio

Corresponding author(s): Søren Brunak

Last updated by author(s): Apr 5, 2022

Reporting Summary

Nature Portfolio wishes to improve the reproducibility of the work that we publish. This form provides structure for consistency and transparency in reporting. For further information on Nature Portfolio policies, see our Editorial Policies and the Editorial Policy Checklist.

Statistics

For all statistical analyses, confirm that the following items are present in the figure legend, table legend, main text, or Methods section.

n/a	Confirmed	
☐	☒	The exact sample size (n) for each experimental group/condition, given as a discrete number and unit of measurement
☐	☒	A statement on whether measurements were taken from distinct samples or whether the same sample was measured repeatedly
☒	☐	The statistical test(s) used AND whether they are one- or two-sided<br>*Only common tests should be described solely by name; describe more complex techniques in the Methods section.*
☒	☐	A description of all covariates tested
☒	☐	A description of any assumptions or corrections, such as tests of normality and adjustment for multiple comparisons
☐	☒	A full description of the statistical parameters including central tendency (e.g. means) or other basic estimates (e.g. regression coefficient) AND variation (e.g. standard deviation) or associated estimates of uncertainty (e.g. confidence intervals)
☒	☐	For null hypothesis testing, the test statistic (e.g. F, t, r) with confidence intervals, effect sizes, degrees of freedom and P value noted<br>*Give P values as exact values whenever suitable.*
☒	☐	For Bayesian analysis, information on the choice of priors and Markov chain Monte Carlo settings
☒	☐	For hierarchical and complex designs, identification of the appropriate level for tests and full reporting of outcomes
☒	☐	Estimates of effect sizes (e.g. Cohen's d, Pearson's r), indicating how they were calculated

Our web collection on statistics for biologists contains articles on many of the points above.

Software and code

Policy information about availability of computer code

Data collection	Python v3.9.7, Pandas v1.1.3, Numpy v1.20.2, R v4.0.0, dplyr v1.0.8
Data analysis	Python v3.9.7, Pandas v1.1.3, Numpy v1.20.2, Keras v2.4.3, Hyperopt v0.2.7, Scipy v1.5.2, Scikit_survival 0.17.1, Scikit_learn 1.0.2

For manuscripts utilizing custom algorithms or software that are central to the research but not yet described in published literature, software must be made available to editors and reviewers. We strongly encourage code deposition in a community repository (e.g. GitHub). See the Nature Portfolio guidelines for submitting code & software for further information.

Data

Policy information about availability of data

All manuscripts must include a data availability statement. This statement should provide the following information, where applicable:

- Accession codes, unique identifiers, or web links for publicly available datasets
- A description of any restrictions on data availability
- For clinical datasets or third party data, please ensure that the statement adheres to our policy

Due to national and EU regulations the data accessed in this paper cannot readily be shared to the wider research community. However, data are made available for use in secure, dedicated environments via application to the Danish Patient Safety Authority and the Danish Health Data Authority.

Field-specific reporting

Please select the one below that is the best fit for your research. If you are not sure, read the appropriate sections before making your selection.

☒ Life sciences ☐ Behavioural & social sciences ☐ Ecological, evolutionary & environmental sciences

For a reference copy of the document with all sections, see nature.com/documents/nr-reporting-summary-flat.pdf

Life sciences study design

All studies must disclose on these points even when the disclosure is negative.

Sample size	This study is based on historical data and we use all available data. It is not meaningful to determine a sample size.
Data exclusions	In this study we retrospectively collected electronic patient record (EPR) data from patients admitted to one of 10 mixed medical and surgical ICUs in the Capital Region of Denmark and the Region of Southern Denmark covering the period 6th of September 2011 through 19th of April 2018. Data were excluded for patients younger than 16 years of age and patients without available outcome data due to missing national identification number (NIN; all Danish citizens receive a NIN at birth or immigration).
Replication	ICU patients were randomly split into a training set and a hold-out test set. Patients born in the first 6 days of a month were assigned to the test set, yielding about 80% for training and 20% for testing.
Randomization	ICU patients were randomly split into a training set and a hold-out test set. Patients born in the first 6 days of a month were assigned to the test set, yielding about 80% for training and 20% for testing.
Blinding	N/A

Reporting for specific materials, systems and methods

We require information from authors about some types of materials, experimental systems and methods used in many studies. Here, indicate whether each material, system or method listed is relevant to your study. If you are not sure if a list item applies to your research, read the appropriate section before selecting a response.

Materials & experimental systems

n/a	Involved in the study	
☒	☐	Antibodies
☒	☐	Eukaryotic cell lines
☒	☐	Palaeontology and archaeology
☒	☐	Animals and other organisms
☒	☐	Human research participants
☐	☒	Clinical data
☒	☐	Dual use research of concern

Methods

n/a	Involved in the study	
☒	☐	ChIP-seq
☒	☐	Flow cytometry
☒	☐	MRI-based neuroimaging

Clinical data

Policy information about clinical studies

All manuscripts should comply with the ICMJE guidelines for publication of clinical research and a completed CONSORT checklist must be included with all submissions.

Clinical trial registration	N/A. This study is not a clinical trial. This study is based on secondary use of historical health data.
Study protocol	N/A. This study is not a clinical trial. This study is based on secondary use of historical health data.
Data collection	N/A. This study is not a clinical trial. This study is based on secondary use of historical health data.
Outcomes	N/A. This study is not a clinical trial. This study is based on secondary use of historical health data.
